# Recognition Times for 54 Thousand Dutch Words: Data from the Dutch Crowdsourcing Project

**DOI:** 10.5334/pb.491

**Published:** 2019-07-17

**Authors:** Marc Brysbaert, Emmanuel Keuleers, Paweł Mandera

**Affiliations:** 1 Department of Experimental Psychology, Ghent University, BE; 2 Department Cognitive Science and Artificial Intelligence, Tilburg University, NL

**Keywords:** word recognition, megastudy, open access

## Abstract

We present a new database of Dutch word recognition times for a total of 54 thousand words, called the Dutch Crowdsourcing Project. The data were collected with an internet vocabulary test. The database is limited to native Dutch speakers. Participants were asked to indicate which words they knew. Their response times were registered, even though the participants were not asked to respond as fast as possible. Still, the response times correlate around .7 with the response times of the Dutch Lexicon Projects for shared words. Also results of virtual experiments indicate that the new response times are a valid addition to the Dutch Lexicon Projects. This not only means that we have useful response times for some 20 thousand extra words, but we now also have data on differences in response latencies as a function of education and age. The new data correspond better to word use in the Netherlands.

## Introduction

Word features are characteristics inherent to words. Therefore, you cannot manipulate them at will (Lewis & Vladeanu, 2006). All you can do is correlate them with processing times. As a result, multiple regression analysis (and to a lesser extent, structural equation modelling) has become an essential part of psycholinguistic research, in addition to factorial designs where small-scale samples of stimuli are selected and matched on a series of control variables (Baayen, Feldman, & Schreuder, 2006; Balota, Cortese, Sergent-Marshall, Spieler, & Yap, 2004; Lewis & Vladeanu, 2006; Liben-Nowell, Strand, Sharp, Wexler, & Woods, 2019).

Regression analysis works best when you have large datasets to work with, because it leads to more robust estimates of the regression weights and their contributions in terms of variance explained (Kelley, & Maxwell, 2003; Maxwell, 2000). As a result, researchers in several languages have invested in the collection of large databases of word processing times (for a list of studies with links to the data, see http://crr.ugent.be/programs-data/megastudy-data-available, reviewed in Mandera, Keuleers, & Brysbaert, in press). English is by far the most researched language. Dutch is not doing badly either, with five big databases of word processing times.

The first study is the Dutch Lexicon Project (Keuleers, Diependaele, & Brysbaert, 2010). In this megastudy, lexical decision times were collected for 14 thousand visually presented, monosyllabic and disyllabic words. About half of the words were inflected forms (plurals, diminutives, verb forms). The second study (called BALDEY) involved lexical decisions to 2,780 auditorily presented words and was published by Ernestus and Cutler (2015). The third study (the Dutch Lexicon Project 2) again collected lexical decision times to visually presented words (Brysbaert, Stevens, Mandera, & Keuleers, 2016b). Now, 30 thousand words were tested, mainly lemmas (i.e., uninflected, base words) without a length restriction. The fourth study was run by Heyman, Van Akeren, Hutchison, and Storms (2016) and used a speeded fragment completion task. Participants were shown letter strings (e.g., f_lm) and had to decide as fast as possible whether the missing letter was *i* or *o*. Data were gathered for 8,240 lemmas. Finally, Cop, Dirix, Drieghe, and Duyck (2017) registered eye movements while participants were reading the Dutch translation of an English detective novel. On the basis of the eye movement data, gaze durations were determined for 5,575 words (both lemmas and inflected forms).

Having access to more than one database is important, because it allows researchers to focus on replicable patterns across studies rather than getting sidetracked by idiosyncrasies of a single dataset (Munafo et al., 2017). In this article we discuss a sixth database of word processing times we have gathered in the last years. It is based on a crowdsourcing study that was set up to know how well Dutch words are known but, as we will see, the response times are of use too.

Keuleers and Balota (2015) defined a crowdsourcing study as a study in which data are collected outside of the traditional, controlled laboratory settings. The Dutch Crowdsourcing Project (DCP) is an internet-based vocabulary test, in which participants had to indicate which words they knew. In order to correct for response bias, one third of the stimuli were non-words and participants were warned that they would be penalized if they responded “word” to the non-word stimuli.

Although the DCP task involves a yes/no decision, it is important to consider the differences with a traditional lexical decision task. First, participants were not told time was an issue. Second, they were not asked to decide between a word and a non-word. They were asked to indicate which words they knew and not to guess if they were unfamiliar with a sequence of letters. Participants did the test outside of a university setting and did it because they wanted to know their Dutch proficiency level. Still, Harrington and Carey (2009) noticed that under these conditions the response times (RTs) can be informative. The best way to test whether this is true for our internet test as well, is to correlate the DCP times with the reaction times collected in the existing megastudies, which are laboratory-based. Statistically, we can expect the worth of the RTs to increase if many participants take part, because averaging over large numbers reduces the noise in the individual observations.

## Method

The vocabulary test on which the present data are based, has been available for several years and is still running (available at http://woordentest.ugent.be/). It started in collaboration with newspapers and the Dutch television, so that we could reach more people than in a typical psychology study. The main goal of the vocabulary test was to get an idea of how well words are known in the population, a variable we called word prevalence (Brysbaert et al., 2016b; Brysbaert, Mandera, McCormick, & Keuleers, 2019).

Per test participants received 67 or 70 words and 33 or 30 nonwords.^1^ At the end of the test, participants received an estimate of their vocabulary size, which was a big motivation for them to take part and to recommend the test to others. The estimate was computed on the basis of the equation: percentage word responses to words minus percentage word responses to nonwords. The yes/no format with guessing correction is an established form of vocabulary testing in the language proficiency literature (Ferré & Brysbaert, 2017; Harrington, & Carey, 2009; Lemhöfer & Broersma, 2012; Meara, & Buxton, 1987). The vocabulary study was started in 2013. Accuracy data were reported in Brysbaert, Keuleers, Mandera, and Stevens (2014), Keuleers, Stevens, Mandera, and Brysbaert (2015), and Brysbaert et al. (2016b).

The exact instructions were (translated): “In this test you get 100 letter sequences, some of which are existing Dutch words and some of which are made-up nonwords. Indicate for each letter sequence whether it is a word you know or not. The test takes about 4 minutes and you can repeat it as often as you want^2^ (you will get new letter sequences each time). If you take part, you consent to your data being used for scientific analysis of word knowledge. Do not say yes to words you do not know, because yes-responses to nonwords are penalized heavily!”

Specific for DCP is that we did not work with a fixed set of words and nonwords (as in a regular vocabulary test), but each test was composed of a random sample of words and nonwords. The words were selected from a set of 54,319 Dutch words compiled over the years. The nonwords were selected from a list of 24,924 pseudowords generated with Wuggy (Keuleers & Brysbaert, 2010). Because of this feature, participants could take the test more than once. Indeed, a few participants took several hundreds of tests over the years.

Further specific to the DCP stimulus set is that the vast majority of words consist of uninflected lemma forms. This is different from DLP, where about half of the stimuli were inflected forms (the only inclusion criterion was monosyllabic or disyllabic words). On the other hand, there is a big overlap with DLP2, which contained 30 thousand lemmas.

Before the start of the test, participants were asked a few basic questions. These were: (1) whether they are native Dutch speakers, (2) where they grew up, (3) what the highest degree is they obtained or are working towards, (4) their gender and age, and (5) how many languages they speak in addition to Dutch and the mother tongue. Participants were not required to provide this information before they could take part, but the vast majority did.

## Results and discussion

The data used in the present article were downloaded in September 2018 and contain all the tests taken between the beginning of project (March 16, 2013) and September 2018. We limit the analyses to the word data of the participants who completed the questions at the beginning and indicated they were native Dutch speakers. Because the test was more popular in Belgium than in the Netherlands, 43% of the data came from people growing up in Belgium and 55% from people growing up in the Netherlands (the population statistics are 28% and 72%).

We considered only responses from the three first sessions associated with each profile (based on the IP address) and only took into account the responses from the 10th and subsequent responses given in the test. Trials 1–9 were considered as training trials although they were not explicitly specified as such in the instructions. This left us with 26 million responses to words from 410 thousand sessions. About 30% of the sessions were collected using devices with touchscreen; the other from keyboard devices.

Per word there are on average 486 observations, going from a minimum of 47 to a maximum of 698. The small numbers come from words added to the list in later stages. Cautious users may want to exclude entries with less than 100 observations from their analyses (N = 1,374), as the RTs are less reliable.

RTs were calculated on correct trials only. RTs were defined as the time interval between the presentation of the stimulus and the response of the participant. Overall accuracy was .84. We performed further basic cleaning to limit the amount of noise. We removed all trials with responses longer than 8,000 ms (to make sure no dictionary could be consulted) and subsequently removed exceedingly fast and slow responses using an adjusted boxplot method for positively skewed distributions (Hubert & Vandervieren, 2008) calculated separately for the words in each individual session. These steps were introduced in 2015 (see Mandera, 2016, Chapter 4) and were calculated automatically in a pipeline of programs we developed to process the data. It results in some 5–7% outliers removed. Importantly, all steps were run before we analyzed the mean word RTs. No post analysis optimization took place, based on a garden of forking paths, which is likely to result in data overfitting. Researchers who have reasons to question the choices we made or who want to increase transparency through a multiverse analysis (Steegen, Tuerlinckx, Gelman, & Vanpaemel, 2016) have access to the raw data on https://osf.io/5fk8d/. We are confident that the choices we made will stand scrutiny.

After applying the cleaning procedure, mean RT was 1,326 ms (SD over stimuli is 282). The mean standard deviation in RTs per stimulus was 752 ms (SD over stimuli is 198). Both values are considerably higher than in laboratory based megastudies. For the lexical decision part of DLP2, mean RT for the words was 600 ms (SD = 79) and mean standard deviation of the LDT latencies was 170 ms (SD = 57).

We also calculated standardized RTs (zRTs) by taking the z-values of each session. This eliminates differences in speed and RT range of individual participants and has been shown to reduce the percentage of noise in DLP and DLP2. We can expect the difference between RT and zRT to be smaller in DCP, because there are many more observations per word (almost 500 against less than 40), and because each participant only contributed a tiny bit of data. Indeed, the correlation between RT and zRT for DCP is .977, against .885 in DLP and .953 in DLP2. As a result, below we only discuss simulations with raw RTs, because they are easier to relate to.

### Correlations with data from other megastudies

A first way to measure the merit of the RTs in DCP is to correlate them with the RTs from the other megastudies. In a first analysis, we limit the stimuli to the words present in DCP, DLP, and DLP2.^2^ For DLP and DLP2 we used standardized RTs (zRTs).

**Figure 1:**
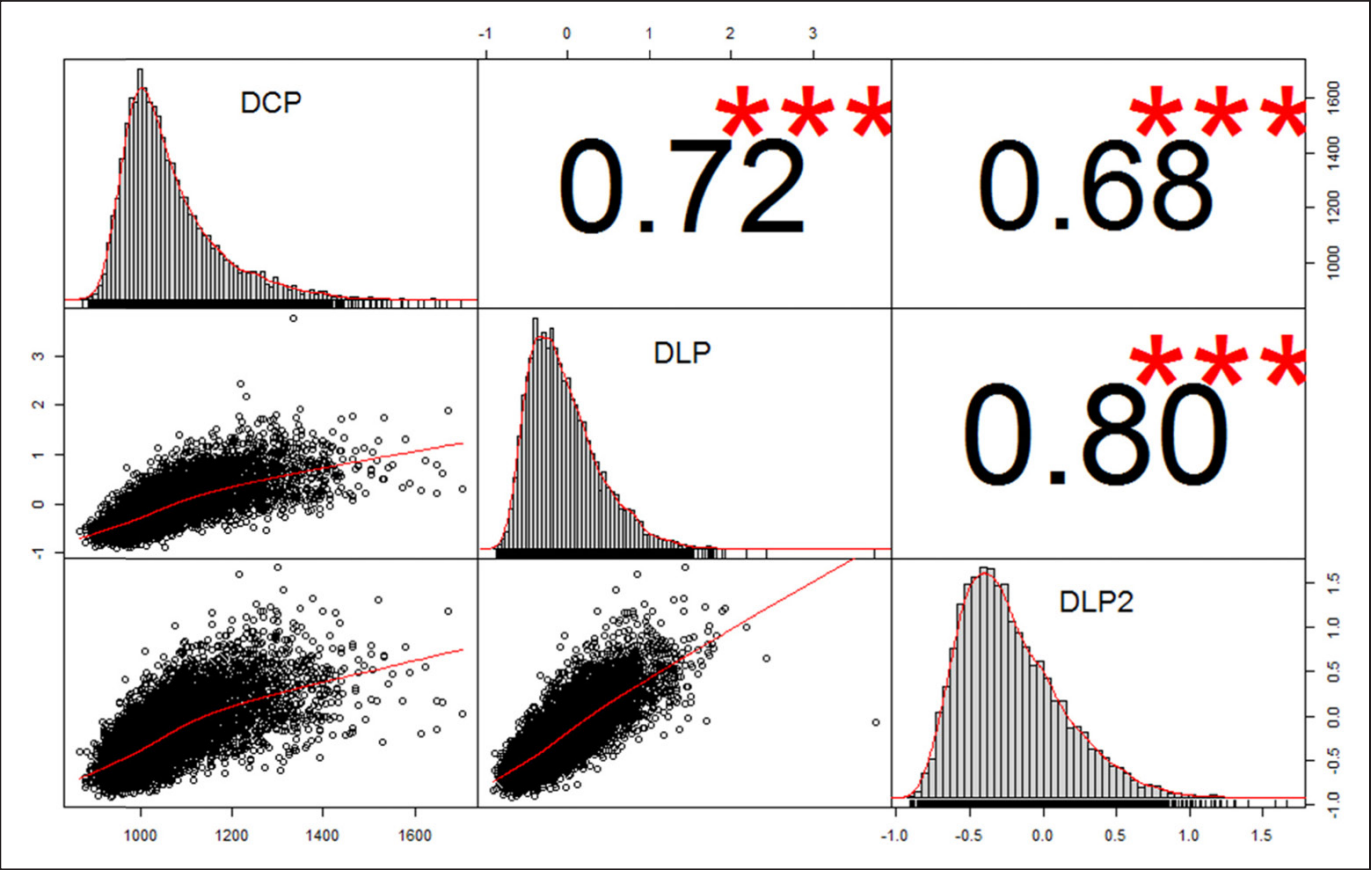
Correlations between the RTs of DCP, DLP, and DLP2 for the items in common that were generally known (N = 7,287). For DLP and DLP2 standardized RTs were used.

We excluded words that had an accuracy of less than .80 in DCP, as the RTs of these words are less trustworthy. This left us with a total of 7,287 words for which we had RTs in all databases. Because of DLP, the observations are limited to monosyllabic and disyllabic words (the words most often used in experimental research). Figure 1 gives the correlations between the databases. As can be seen, the correlation between DLP and DLP2 is higher than between DCP and DLP or DLP2. This is different from a similar set of data we analyzed in English, where the correlation between the English Crowdsourcing Project and the English Lexicon Project was .8 (Mandera et al., in press).

A first factor that seems to contribute to the reduced correlation between DCP and DLP/DLP2 is that the relationship between DCP and DLP/DLP2 has a non-linear component (see Figure 1). The contribution of this factor is very small, however. When an extra predictor is added to capture the nonlinearity, the percentages of variance accounted for increase by .1% only.

Another reason for the lower than expected correlation between DCP and DLP/DLP2 could be that DLP and DLP2 were collected in Belgium, whereas DCP was mainly collected in the Netherlands. To test this possibility, we split DCP in Belgium and the Netherlands. The former had on average 206 observations per word; the latter 267. As can be seen in Table 1, the correlation with data from the Netherlands was lower indeed. However, the correlation with the data from Belgium did not improve, arguably because of the smaller number of observations. Indeed, the most important reason why the data were better for the English Crowdsourcing Study than for DCP probably is that we had on average 666 observations per word in the former study, against 486 observations in the present study.

**Table 1:** Correlations between the RTs of DCP_Belgium_, DCP_Netherlands_, DLP, and DLP2 for the items in common that were generally known (N = 7,287). For DLP and DLP2 standardized RTs were used.

	DLP	DLP2

DCP	.72	.68
DCP_BE_	.71	.67
DCP_NL_	.65	.61
DLP		.80

All in all, Figure 1 and Table 1 show that some 70% of the variance in DCP, DLP, and DLP2 is systematic variance that can be accounted for by word features.

A second way to examine the usefulness of the DCP RTs is to see how well they correlate with the RTs of each of the existing megastudies and, in particular, how they compare to the full DLP2 dataset. Table 2 lists the findings. The table gives some further evidence for the hypothesis that country differences contribute to the reduced correlation between DCP and DLP2. All databases correlate more with DLP2 than with DCP, except for BALDEY, which was collected in the Netherlands (Nijmegen). At the same time, DLP2 for most datasets remains superior to DCP_BE_ and splitting the observations largely offsets any gain observed as a result of using a country-specific measure. So, for most purposes, the aggregate DCP value is to be preferred to DCP_BE_ and DCP_NL_.

**Table 2:** Correlation of the DCP and DLP2 data with other datasets (zRTs for DLP and DLP2). Between brackets the number of shared items.

	N_stimuli_	DCP	DCP_BE_	DCP_NL_	DCPL2

DLP	14,089	.68 (9,131)	.68 (9,131)	.64 (9,131)	.79 (7,503)
BALDEY	2,780	.42 (1,499)	.36 (1,499)	.44 (1,499)	.29 (1,160)
DLP2	30,016	.71 (29,937)	.71 (29,937)	.65 (29,937)	—	
Fragment	8,240	.33 (3,117)	.33 (3,117)	.30 (3,117)	.40 (2,731)
GECO	5,575	.29 (3,519)	.27 (3,519)	.29 (3,519)	.32 (3,108)

### Variance accounted for by word characteristics

A third way to gauge the quality of the DCP dataset is to see how strongly RTs are influenced by word characteristics. In a recent article, Brysbaert et al. (2016b) evaluated the contribution of seven variables to DLP2 zRTs.^3^ They were:

– Word frequency (SUBTLEX-NL expressed as Zipf-scores, which are logarithmic scores going from 1 to 7, with 1 being very low-frequency words and 7 being very high-frequency words; Brysbaert, Mandera, & Keuleers, 2018; Keuleers, Brysbaert & New, 2010)
– Word length (in letters)
– Word length (in syllables)
– Dominant Part of Speech (verb, noun, adjective/adverb, function word, number word)
– Orthographic distance to other words (Yarkoni, Balota, & Yap, 2008)
– Age of acquisition (Brysbaert, Stevens, De Deyne, Voorspoels, & Storms, 2014)
– Concreteness (Brysbaert et al., 2014)

**Figure 2:**
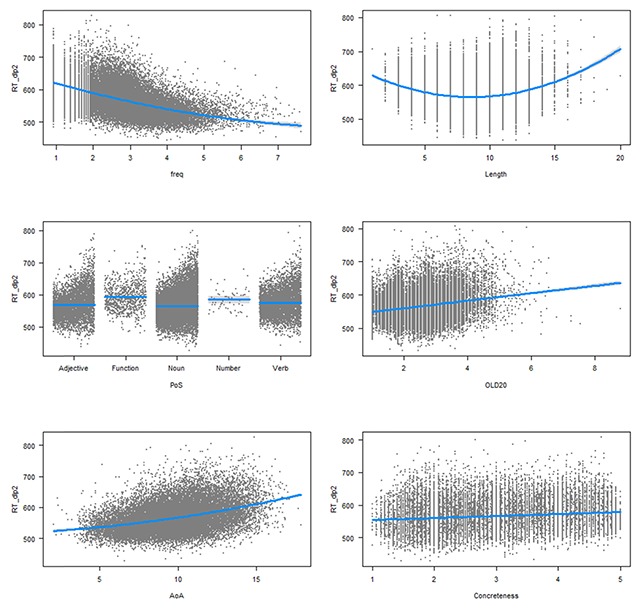
Effects of the variables on the DLP2 lexical decision times. First line: effects of word frequency and length in letters; second line: Part of speech and orthographic distance to other words; third line: age of acquisition and concreteness. The nonsignificant effect of syllable length is not shown.

**Figure 3:**
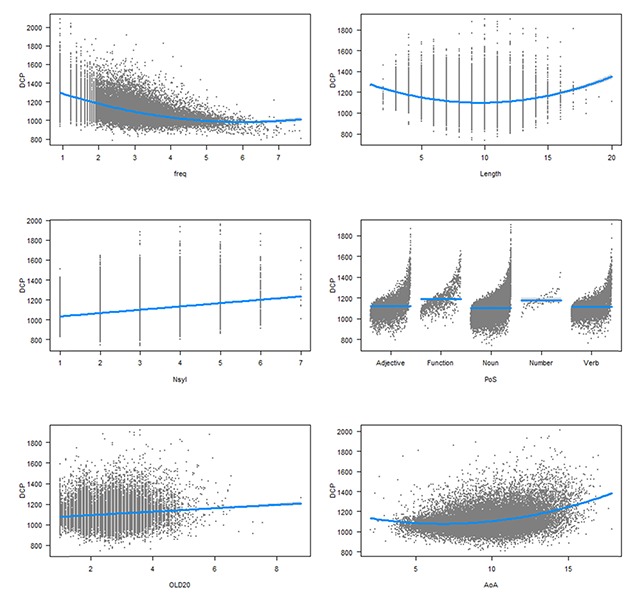
Effects of the variables on the DCP word recognition times. First line: effects of word frequency and length in letters; second line: number of syllables and part of speech; third line: orthographic distance to other words and age of acquisition. The non-significant effect of concreteness is not shown.

Table 3 compares the regression analysis for the words in common between DLP2 and DCP with an accuracy in DLP2 above 66.6% (in order to exclude RTs for unknown words) and for which we had information about the various word characteristics (N = 24,560). To ease the comparison, beta coefficients are given. For these the dependent and independent variables are standardized, so that the coefficients have the same interpretation. Figures 2 and 3 give a graphical display of the effects (based on the raw RTs).

**Table 3:** Outcome of regressions on the DLP2 and DCP RTs for the words in common (N = 24,560). In order to ease the comparison, beta coefficients are given, which have the same meaning for both regressions. Predictors are centered. PoS coefficients are relative to adjective/adverb.

	DLP2	DCP

Word frequency	–.42***	–.46***
Word frequency squared	.05***	.10***
Word length (letters)	.02*	–.04***
Word length (letters) squared	.15***	.12***
Number of syllables	–.01	.19***
PoS_function word_	.08***	.09***
PoS_noun_	–.03***	–.05***
PoS_number word_	.01**	.02***
PoS_verb_	.04***	–.00
OLD	.15***	.07***
AoA	.31***	.22***
AoA squared	.03***	.12***
Concreteness	.12***	–.01
R^2^ =	.43	.49

*** p < .001, ** p < .01, * p < .05.

As can be seen in Table 3 and Figures 2 and 3, the effects of the word variables were quite comparable in DLP2 and DCP. High frequency words were responded to faster than low frequency words, except for the very high-frequency words, which are mostly function words (pronouns, determiners, prepositions, auxiliaries, particles). Words with 8–9 letters were responded to most rapidly. Words with more syllables were responded to more slowly in DCP but not in DLP2. Function words and number words took longer to respond to than content words, possibly because they are rarely seen in isolation. Indeed, the processing costs for these words are not observed in eye movement studies (Dirix, Brysbaert, & Duyck, in press). Words that were orthographically more distant to other words took more time to respond to, in line with the proposal that speeded responses in a lexical decision task are not always based on individual word recognition but can be based on the total degree of orthographic activation caused by the letter string (Grainger & Jacobs, 1996; Pollatsek, Perea, & Binder, 1999). Words that are similar to other words create more initial activation in the lexicon. Orthographic distance had a stronger effect in DLP2 than in DCP, in line with the fact that responses in DLP2 were more time pressured. Late acquired words took longer to respond to than early acquired words, both in DCP and DLP2. Finally, concreteness had an unexpected effect in DLP2 (concrete words took longer to respond to than abstract words) and no effect in DCP.

All in all, the similarities between DCP and DLP2 are larger than the differences. The percentage of variance accounted for was larger in DCP (R^2^ = 49) than in DLP2 (R^2^ = .43). This is lower than the correlation between the datasets (r = .71), meaning we are still missing some 20–25% of systematic variance in RTs that can be accounted for.

### Virtual experiments

A final way to probe the value of DCP is to see whether we can replicate some classic studies with the dataset. Keuleers et al. (2010) ran a number of virtual experiments with DLP. The first study they tried to replicate was Schreuder and Baayen (1997). These authors addressed the question to what extent lexical decision times to singular nouns are influenced by the frequencies of the plurals. For instance, the words spier (muscle) and stier (bull) have more or less the same frequency, but the plural form spieren (muscles) occurs significantly more often than the plural form stieren (bulls). Schreuder and Baayen hypothesized that singular nouns with frequent plurals would be responded to faster than matched singular nouns with non-frequent plurals. After confirming this hypothesis, they examined the effect of the number of morphologically related nouns (family size) and the cumulative frequency of all family members (cumulative frequency). All in all, Schreuder and Baayen ran five experiments. Table 4 shows the original results, together with the outcome of virtual experiments in DLP, DLP2, and DCP. The effects are replicated in all databases, including DCP.

**Table 4:** Reaction times (in ms) to singular Dutch nouns as a function of the frequencies of the plurals and the family size, as reported by Schreuder and Baayen (1997) and in virtual experiments. Means and significance based on item analysis.

	Original	DLP	DLP2	DCP

*Exp 1*			

High-frequency plurals	539	579	554	1026
Low-frequency plurals	578	619	525	992
Difference	39**	40**	29**	34*
***Exp 2***			

High cumulative family frequency	594	601	546	1045
Low cumulative family frequency	650	652	597	1112
Difference	56**	51**	51**	67*
***Exp 3***			

High family size	553	584	542	1004
Low family size	594	638	572	1070
Difference	41*	54**	30*	66*
***Exp 4 (family size fixed)***			

High cumulative frequency	632	651	580	1098
Low cumulative frequency	632	644	571	1046
Difference	0	–7	–9	–52
***Exp 5 (family size and cumulative frequency fixed)***			

High frequency word	577	618	570	1046
Low frequency word	674	674	629	1209
Difference	97**	56*	59**	163**

* p < .05, ** p < .01 (in analysis over items).

A second topic Keuleers et al. (2010) addressed, was how cognates are processed. Cognates are words with similar form and meaning in two languages (e.g., the Dutch words film [film] and appel [apple]). Bilinguals have a processing advantage for cognates relative to matched controls. Van Hell and Dijkstra (2002) reported that Dutch native speakers responded about 30 ms faster to Dutch–English cognates in a lexical decision task than to control words. Interestingly, the effect was much smaller for Dutch–French cognates, arguably because Dutch speakers from the Netherlands have a larger knowledge of English. To test the latter hypothesis, van Hell and Dijkstra (2002) tested bilinguals with a high proficiency in French (these were students taking a French degree), and found more evidence for a French cognate effect. Surprisingly, for the highly proficient French speakers, the English cognate effect was also larger.

Table 5 shows the findings of the original study and virtual experiments in DLP, DLP2, and DCP. Although the findings are replicated in all studies, the English cognate effect failed to reach statistical significance in DCP.

**Table 5:** The cognate effect reported by van Hell and Dijkstra (2002). Left part: original data. Right part: virtual experiments. Data and statistics based on item means.

	Original Low French	Original High French	DLP	DLP2	DCP

Dutch–English cognates	499	489	553	511	974
Dutch–French cognates	519	520	579	522	1019
Control words	529	541	586	534	1012
English cognate effect	30*	52**	33*	23*	38
French cognate effect	10	21*	7	12	–7

A third issue examined by Keuleers et al. (2010) was the age-of-acquisition (AoA) effect. Brysbaert, Lange, and Van Wijnendaele (2000) published a series of experiments showing that a word frequency effect was still found when words are controlled for length, AoA, and imageability. Similarly, a significant AoA effect was found when all other variables were controlled for. However, no significant effect of imageability was found once the stimuli were controlled for length, frequency, and AoA. Table 6 lists the original findings (left part). As the right part of the table shows, the same findings are obtained in the virtual experiments as in the original study.

**Table 6:** The effects of AoA, frequency, and imageability reported by Brysbaert et al. (2000). Left part: original data. Right part: virtual experiments. Data and statistics based on item means.

	Original	DLP	DLP2	DCP	

***AoA***				

Early	594	580	537	993
Late	646	638	584	1080
Effect	52**	58**	47**	87**
***Frequency***				

High	554	550	512	970
Low	639	631	563	1103
Effect	85**	81**	51**	133**
***Imageability***				

High	609	597	531	1013
Low	609	608	549	1057
Effect	0	11	18	44

A final study Keuleers et al. (2010) sought to replicate, was van Hell and de Groot (1998). These authors argued that imageability/concreteness does not have a genuine effect in lexical decision but that it is a context availability (CA) effect in disguise. CA indicates how easily a participant can think of a context in which the word can be used. To investigate the issue, van Hell and de Groot (1998) compiled four lists of 20 words. The first two lists compared abstract and concrete words that were matched on CA; the second two compared abstract and concrete words confounded for CA (i.e., the CA was higher for the concrete than the abstract words). Only in the latter condition did van Hell and de Groot (1998) find a significant difference (see the left part of Table 7), making them conclude that the concreteness effect was a CA effect in disguise. As before, the same conclusion is reached on the basis of the virtual experiments (right part).

**Table 7:** The effects of concreteness and context availability (CA) reported by van Hell and de Groot (1998). Left part: original data. Right part: virtual experiments. Data and statistics based on item means.

	Original	DLP	DLP2	DCP	

***Matched on CA***				

Abstract	541	560	519	980
Concrete	554	572	525	992
Difference	–13	–12	–6	12
***Confounded with CA***				

Abstract	554	573	515	1001
Concrete	523	536	508	966
Difference	31**	37**	7	35**

All in all, DCP seems to replicate basic findings in Dutch word recognition research as well as DLP and DLP2. The effects tend to be a bit larger in terms of ms difference, in line with the longer response times of DCP. At the same time, it looks like DCP contains more noise than DLP and DLP2, requiring a few more stimuli per condition to obtain significant effects. As Brysbaert and Stevens (2018) argued, a good word recognition experiment has at least 40 stimuli per condition, a criterion not met in most of the studies discussed above.

### Education differences

Up to now we have discussed findings DCP has in common with DLP and DLP2 and seen that for these words DCP is a valid addition to the existing megastudies. However, the merit of DCP goes further. For a start, DCP offers data for 20 thousand words not covered by DLP2, and for 35 thousand words not present in DLP. This substantially increases the resources available to researchers.

In addition, DCP includes more participants than the typical undergraduate students. Some participants had only finished high school, others had achieved a bachelor degree (often outside university), or a master degree (at university). On average, we had 135 observations per word for participants who finished high school, 175 for participants with a bachelor degree, and 160 for participants with a master degree.

Keuleers et al. (2015) and Brysbaert, Stevens, Mandera, & Keuleers (2016a) already discussed the number of words known as a function of education level. Participants with more education know more words than participants with less education. Interestingly, the differences were modest when the participants’ age was taken into account and mainly originated during the study years, arguably because the participants then were acquiring the academic vocabulary related to their studies and word use in higher education (Coxhead, 2000).

To compare the three education groups, we report the outcome of the regression analysis with the variables discussed in Table 3. Two outcomes are given: First, the analysis with the raw regression weights, and then the analysis with the beta coefficients. The former tells us how the RTs differ between groups, the latter how the relative importance of the variables varies. We limit the analysis to the words known by at least 80% of the DCP participants and for which we have all data (N = 26,523). To ease the comparison of the regression weights, predictors were centered.

Table 8 shows the outcome of the analyses. Participants with less education responded slightly more slowly as can be seen in the intercepts, but for the rest do not show strong differences. Interestingly, R^2^ was lower for the participants with a master degree than for those with a high school degree. It is not clear what the origin is of this drop.

**Table 8:** Outcome of regressions on the DCP RTs for the different education groups. Analysis limited to the words known by more than 80% of the participants for which we have all data (N = 26,523). Both regression weights and beta coefficients are given. Predictors are centered.

	DCP_High_	DCP_Bach_	DCP_Mast_

***Regression weights***			

Intercept	1156***	1107***	1094***
Word frequency	–75***	–74***	–71***
Word frequency^2^	10***	10***	10***
Word length (letters)	–5***	–4***	–3***
Word length (letters)^2^	3***	2***	2**
Number of syllables	37***	24***	14***
PoS_function word_	66***	75***	76***
PoS_noun_	–22***	–17***	–16**
PoS_number word_	61***	63***	70***
PoS _verb_	–2	3	8**
OLD	22***	15***	11***
AoA	23***	18***	14***
AoA^2^	4***	2***	1***
Concreteness	–1	–1	6***
R^2^ =	.505	.454	.381
***Beta coefficients***			

Word frequency	–.40	–.45	–.46
Word frequency^2^	.08	.09	.10
Word length (letters)	–.07	–.06	–.05
Word length (letters)^2^	.12	.12	.13
Number of syllables	.20	.15	.10
PoS_function word_	.07	.08	.09
PoS_noun_	–.06	–.05	–.05
PoS_number word_	.02	.02	.02
PoS_verb_	–.00	.01	.02
OLD	.11	.08	.07
AoA	.30	.26	.23
AoA^2^	.14	.09	.04
Concreteness	–.01	.01	.04

*** p < .001, ** p < .01, * p < .05.

**Figure 4:**
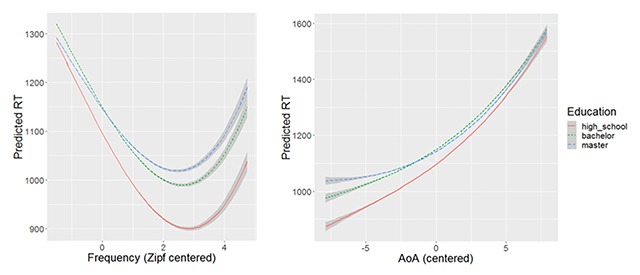
Predicted response times for the three education groups as a function of word frequency and AoA. Model as specified in Table 8.

Figure 4 shows how the predicted RTs differ for the three education groups as a function of word frequency and AoA. Whereas the frequency effect is very similar for the three education groups, the AoA effect is larger for the less educated, arguably because they do not know (well) the words that are typically acquired late (e.g., as part of university education).

### Age differences

Another variable we can look at, is the age group of the participants. Davies, Arnell, Birchenough, Grimmond, and Houlson (2017) reported that the effects of word frequency and AoA on lexical decision times become smaller with increasing age over adult life. At the same time, there was ageing-related response slowing, which could be attributed to decreasing efficiency of stimulus encoding and/or response execution processes in older age. Alternatively, since more exposure to language increases the vocabulary of a person (Keuleers et al., 2015; Verhaeghen, 2003), response slowing is also consistent with increased processing costs related to the accumulation of information over time (Ramscar, Hendrix, Shaoul, Milin, & Baayen, 2014).

A number of studies have demonstrated that the word frequency effect is expected to become smaller with growing language exposure (Brysbaert, Lagrou, & Stevens, 2017; Brysbaert, Mandera, & Keuleers, 2018; Cop, Keuleers, Drieghe, & Duyck, 2015; Diependaele, Lemhöfer, & Brysbaert, 2013; Mainz, Shao, Brysbaert, & Meyer, 2017; Mandera, 2016, Chapter 4; Monaghan, Chang, Welbourne, & Brysbaert, 2017). This finding is also consistent with connectionist models, which show a decrease in the frequency effect when overlearning takes place (Monaghan et al., 2017) and with the assumption that word learning follows a power law rather than an exponential law (Logan, 1988; Mandera, 2016, Chapter 4).

In contrast to the above work, Cohen-Shikora and Balota (2016) did not observe a decrease in the word frequency effect as a function of age in lexical decision, word naming, and animacy judgment. Still, they replicated some of the core effects of the other studies: (1) Older participants were slower and more accurate than younger participants, (2) older participants had a larger vocabulary than younger participants, and (3) there was a negative correlation between vocabulary size and the word frequency effect.

To test the age differences, we made a distinction between participants of 18–29 years (on average 133 observations per word), 30–49 (169 observations), and 50+ (147 observations).

**Figure 5:**
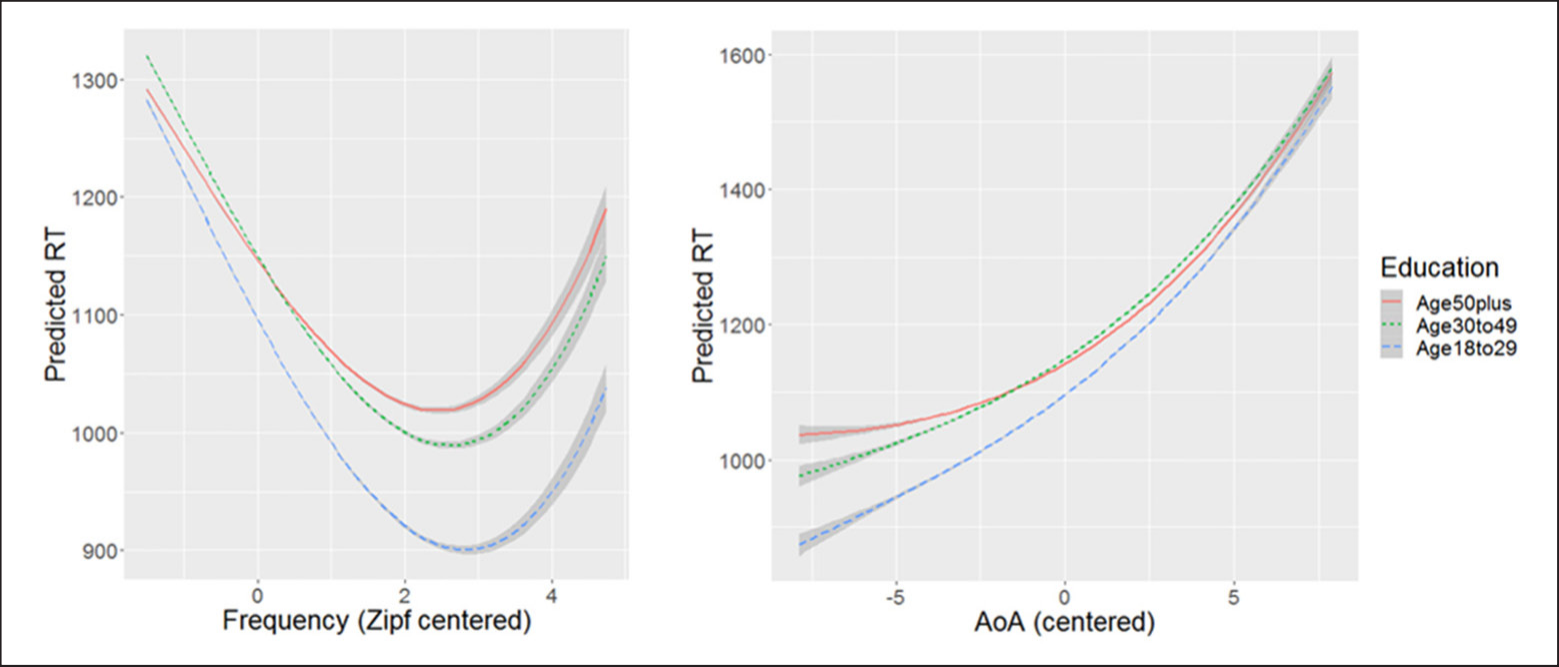
Predicted response times for the three age groups as a function of word frequency and AoA.

Table 9 and Figure 5 show the results of the regression analysis. Younger participants are faster for the easy words (early acquired, high frequency) but not for the difficult words (late acquired, low frequency), in line with patterns reported by Davies et al. (2017) and ourselves, and counter to Cohen-Shikora and Balota (2016). Another clear effect is that older participants seem to require more time for extra syllables. Both patterns were also observed in the comparable English Crowdsourcing Project (Mandera et al., in press).

**Table 9:** Predicted response times for the three education groups as a function of word frequency and AoA.

	DCP_18-29_	DCP_30-49_	DCP50_+_

*Regression weights*			

Intercept	1079***	1133***	1128***
Word frequency	–84***	–75***	–60***
Word frequency^2^	10***	11***	9***
Word length (letters)	–2**	–4***	–6***
Word length (letters)^2^	2***	3***	3***
Number of syllables	16***	25***	30***
PoS_function word_	84***	72***	60***
PoS_noun_	–14***	–18***	–20***
PoS_number word_	82***	64***	55***
PoS_verb_	–0	4	8**
OLD	13***	15***	19***
AoA	21***	17***	15***
AoA^2^	2***	2***	2***
Concreteness	3*	2	3**
R^2^ =	.446	.437	.408
***Beta coefficients***			

Word frequency	–.46	–.45	–.39
Word frequency^2^	.09	.10	.09
Word length (letters)	–.03	–.06	–.09
Word length (letters)^2^	.11	.13	.14
Number of syllables	.09	.15	.20
PoS_function word_	.09	.08	.07
PoS_noun_	–.04	–.06	–.07
PoS_number word_	.02	.02	.02
PoS_verb_	–.00	01	.02
OLD	.07	.08	.11
AoA	.28	.25	.24
AoA^2^	.08	.08	.11
Concreteness	.02	.01	.02

*** p < .001, ** p < .01, * p < .05.

## Conclusions

We present a new word database, the Dutch Crowdsourcing Project (DCP), which is larger than the available datasets. It is larger both in the number of words included and in the variety of participants taking part.

The database was collected by means of an internet vocabulary test, in which participants indicated which words they know and which not. In order to discourage yes responses to unknown words, about one third of the stimuli were nonwords and participants were penalized if they said yes to these nonwords. We collected 26 million responses to words.

Although speed of responding was not mentioned as an evaluation criterion to the participants, the present analyses show that the response times correlate well with lexical decision times collected in laboratory settings, although they are some 450–500 ms longer. This suggests that the main bulk of the extra time in DCP is unrelated to word recognition itself (see Ratcliff, Gomez, & McKoon, 2004, for a model that includes such a time component). The longer response times led to slightly larger effects in the virtual experiments but with less power (due to the higher variability in the data). The latter can be compensated for by including more stimuli in the analysis.

To some extent it is surprising that untimed answers to a vocabulary test resemble lexical decision times so well, when based on large numbers of observations. This testifies to the ecological validity of the lexical decision task, as very much the same results are obtained in an untimed vocabulary test outside of academia as on a speeded response task in the laboratory.

DCP is further interesting because a large range of people took part. Surprisingly, we found no big differences between education levels (Figure 3). Presumably this is due to the fact that only people interested in language took part in the test. There is evidence, for instance, that the size of the frequency effect depends more on the amount of reading and language exposure than on the intelligence or the education level of the participants (Brysbaert et al., 2017). DCP does point to some interesting effects of age (or language exposure), however. The effects of frequency and age of acquisition seem to become smaller as adults grow older (see also Davies et al., 2017; but see Cohen-Shikora & Balota, 2016), whereas older people seem to be more affected by the complexity of the word (the number of syllables). Further, targeted experiments will have to confirm these initial impressions.

Part of the variability in RTs is due to country differences (Belgium versus the Netherlands). However, these difference do not outweigh the fact that the number of observations per country is halved. Therefore, researchers will have the least noise in measurements when they use the entire DCP dataset rather than DCP_BE_ or DCP_NL_. If they are concerned about country effects, they can limit the analysis to words with similar prevalence in Belgium and the Netherlands (Brysbaert et al., 2016b).

## Availability

The raw data and the Excel files on which the above analyses are based, are available at the Open Science Framework webpage https://osf.io/5fk8d/ or on our website http://crr.ugent.be/. To facilitate analyses of the full dataset, we release a Python module for working with the raw data (available at https://github.com/pmandera/vocab-crowd).

**Figure 6:**
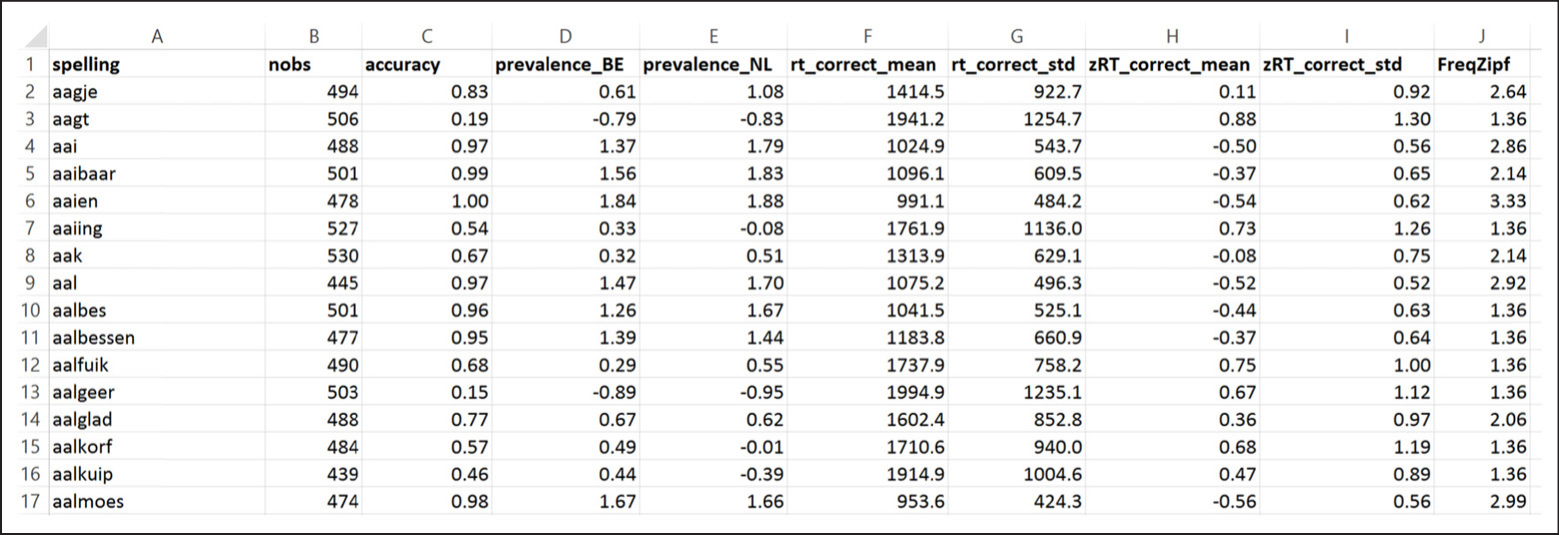
Outline of the DCP master file including RTs based on all native speakers.

The Excel files are for researchers who want easy access to the item data. One of these is the master file containing the information calculated across all participants, called *Dutch Lexicon Project All Native Speakers*. Its outline is shown in Figure 6.

Column A gives the word. Column B says how many observations there were for that word. Column C gives the response accuracy, indicating the number of observations on which the RTs are based. We would prefer users not to use the information of Column C for anything other than the analysis of RTs. In Brysbaert et al. (2016b) we present the word prevalence measure, which is better than accuracy and based on more observations. Word prevalence is given separately for Belgium and the Netherlands in Columns D and E, so that users can target stimulus words at their audience. Columns F to I contain the new information: the DCP RTs and the standard deviations seen across participants, and the same information for the standardized RTs. Finally, for the user’s convenience, Column J includes the SUBTLEX-NL frequencies expressed as Zipf values (Brysbaert et al., 2018).

In addition to the master file, we have an Excel file with the data split per education level (DCP Education levels) and a file per age (DCP Age groups). Users who want other summary files, are invited to make them themselves on the basis of the raw data.

The data can be used freely for research purposes under the Creative Commons’ license Attribution-NonCommercial-ShareAlike (CC BY-NC-SA). They cannot be used in commercial products without written agreement of the authors.

The analyses reported in this paper can be repeated by running the R script at the Open Science Framework webpage. This makes use of two other summary tables that are also made available.

## Footnotes

We started with 67 words and 33 nonwords. Based on the input we received from users, we had several updates in which we pruned the bad words and nonwords. In 2015 we also changed the number of words to 70 and the number of nonwords to 30. This did not make any perceptible difference to the participants and allowed us to collect slightly more word data.

Including the other databases too much reduces the number of stimuli in common.

The word prevalence variable cannot be tested here, because it is based on the same dataset.
